# New Imaging Parameters to Predict Sudden Cardiac Death in Chagas Disease

**DOI:** 10.3390/tropicalmed5020074

**Published:** 2020-05-08

**Authors:** Renata J. Moll-Bernardes, Paulo Henrique Rosado-de-Castro, Gabriel Cordeiro Camargo, Fernanda Souza Nogueira Sardinha Mendes, Adriana S. X. Brito, Andréa Silvestre Sousa

**Affiliations:** 1 D’Or Institute for Research and Education (IDOR), Rio de Janeiro 22281-100, Brazil; renata.moll@idor.org (R.J.M.-B.); paulo.rosado@idor.org (P.H.R.-d.-C.); gabriel.camargo@idor.org (G.C.C.); adriana.soares@idor.org (A.S.X.B.); 2 Institute of Biomedical Sciences, Federal University of Rio de Janeiro (UFRJ), Rio de Janeiro 21941-901, Brazil; 3 Evandro Chagas National Institute of Infectious Diseases, Oswaldo Cruz Foundation, Rio de Janeiro 21040-900, Brazil; fernanda.sardinha@ini.fiocruz.br

**Keywords:** Chagas disease, cardiomyopathy, myocardial fibrosis, myocardial sympathetic denervation, inflammation, sudden death, cardiac magnetic resonance, radionuclide imaging, SPECT-CT, PET-CT

## Abstract

Chronic Chagas’ cardiomyopathy is the most severe and frequent manifestation of Chagas disease, and has a high social and economic burden. New imaging modalities, such as strain echocardiography, nuclear medicine, computed tomography and cardiac magnetic resonance imaging, may detect the presence of myocardial fibrosis, inflammation or sympathetic denervation, three conditions associated with risk of sudden death, providing additional diagnostic and/or prognostic information. Unfortunately, despite its high mortality, there is no clear recommendation for early cardioverter-defibrillator implantation in patients with Chagas heart disease in the current guidelines. Ideally, the risk of sudden cardiac death may be evaluated in earlier stages of the disease using new image methods to allow the implementation of primary preventive strategies.

## 1. Introduction

Chronic Chagas’ cardiomyopathy is the most severe and frequent manifestation of Chagas disease and occurs in 25–30% of infected people. Patients may develop severe clinic manifestations, such as congestive heart failure, malignant arrhythmias or thromboembolism [1,2].

The disease is endemic in Latin America, where it is responsible for a high social and economic burden, however, due to the migration of affected individuals and the increased number of patients infected through other forms of transmission, such as blood transfusion, organ transplantation or vertically from mother to infant, the disease has become a global health concern. Non-endemic countries have reported increasing numbers of patients and are particularly worried about the limited awareness in medical community [3]. Cardiac involvement is the main cause of death. The clinical course of Chagas heart disease (CHD) is variable, and the identification of patients at risk for death remains a challenge [4].

The pathogenesis of chronic CHD involves a progressive inflammatory process and adverse immune response with myocardial necrosis and damage to the conduction tissue, leading to electrocardiographic changes, such as atrioventricular and intraventricular blocks, sinus node dysfunction, ventricular arrhythmias and sudden cardiac death. The presence of myocardial inflammation, necrosis and fibrosis may result in left ventricular (LV) segmental wall motion abnormalities and congestive heart failure. Derangements in the coronary microcirculation are likely to cause ischemic-like symptoms, electrocardiographic (ECG) abnormalities and perfusion defects [5].

Patients with CHD are classified according to symptoms and presence of myocardial abnormalities. The presence of typical ECG abnormalities usually defines the diagnosis of chronic CHD (Table 1). The most common initial findings are conduction disorders, and/or ventricular arrhythmias [6].

Chronic CHD carries a worse prognosis when compared to ischemic and other non-ischemic causes of heart failure [8]. The presence of chronic persistent myocardial inflammation plays a central role in the genesis of arrhythmias, due to irreversible cell damage, fibrosis and scar formation. Ventricular arrhythmias are a major cause of morbidity and mortality in patients with Chagas disease, and may occur even before significant LV systolic dysfunction, leading to sudden cardiac death (SCD) [9,10]. In addition, active inflammation may increase the automaticity within inflamed areas and act as a trigger for reentry in the presence of fibrosis. Besides fibrosis and inflammation, autonomic dysfunction can also be related to the genesis of ventricular arrhythmias.

Identifying patients with chronic CHD at increased risk of SCD is crucial, as they could benefit from prophylactic implantation of cardioverter-defibrillators [11,12,13]. ECG, 24 h Holter and exercise test are extremely useful to diagnose and manage these patients. In addition, new imaging modalities such as strain echocardiography, nuclear medicine and cardiac magnetic resonance (CMR) imaging with late gadolinium contrast enhancement may detect the presence of myocardial fibrosis, inflammation or sympathetic denervation, providing additional diagnostic and/or prognostic information. Acute Chagas disease will not be analyzed here.

## 2. ECG/Holter Monitoring/Exercise Test

### 2.1. Eletrocardiogram (ECG)

Electrocardiogram (ECG) is the most important exam to characterize in which phase Chagas disease patient is. It is an easy method to perform, widely available worldwide and with high sensitivity. If the ECG remains normal, the prognosis of those patients is similar to the general population. It is important to perform ECG on a serial basis annually to assess possible disease progression. Asymptomatic infected patients with normal ECG (chronic indeterminate form) should be followed annually with clinical evaluation and ECG. In the presence of symptoms and/or ECG abnormalities, patients should be ideally submitted to 24-h Holter, exercise test and echocardiography. Usually, the ECG is the first exam to undergo changes when there is cardiac involvement in the chronic phase of Chagas disease. The presence of typical ECG abnormalities is associated with increased risk of progression to more severe cardiomyopathy.

There are some unspecific findings in ECG that need individualized evaluation, as they alone are not sufficient to diagnose cardiac involvement such as: sinus bradycardia, low voltage QRS, incomplete right bundle branch block, anterosuperior hemiblock, first-degree atrioventricular block and unspecific ST-T findings. The most commons early findings are right bundle branch block with or without left anterior fascicular block, second- and third-degree atrioventricular block. Other alterations associated with CHD are complex ventricular arrythmias, atrial fibrillation, complete atrioventricular block and left brunch block. ECG changes associated with worse prognosis are frequent premature ventricular contractions (PVCs), increased QT-interval dispersion, low-voltage QRS, QRS fragmentation, polymorphous or repetitive non-sustained ventricular tachycardia and prolonged QRS.

When an electrocardiographic alteration compatible with Chagas’ disease is identified, the patient is reclassified from chronic undetermined benign form to a cardiac form with a poorer prognosis and additional cardiac and gastrointestinal check-up is recommended (Table 1) [6].

### 2.2. 24 h Holter

A 24 h Holter is recommended when available and in patients with symptoms suggestive of cardiac arrhythmias, such as palpitations, presyncope and syncope, or ECG abnormalities like bradyarrhythmia, second degree atrioventricular block and multiple PVCs. Often, brady and tachyarrhythmias are identified in the same patient, and this differential diagnosis is important for indicating a pacemaker implant associated or not with the implantable cardioverter defibrillator (ICD). A 24 h Holter is useful to identify an increased risk of sudden cardiac death and unmask signs of autonomic dysfunction, such as reduced heart rate variability. The presence of non-sustained ventricular tachycardia on the 24 h Holter is one criterion that worsens the prognosis of patients with CHD. Major electrocardiographic changes are sinus node dysfunction, atrioventricular block, or frequent PVCs that becomes more complex and frequent with the disease progression. The presence of ventricular tachycardia is associated with left or global ventricular dysfunction and, when sustained, configures a worse prognosis. The main cause of sudden death in CHD is ventricular fibrillation, which is more frequent when there are previous episodes of ventricular tachycardia, but it can be the first manifestation of the disease or its terminal event in patients with severe ventricular dysfunction and heart failure. CHD is one of the most common indications of artificial pacemaker implants in Brazil, and a 24 h Holter is important to help the clinician to confirm the need of the device, indicate the best type and the follow-up after the implant, as this can detect faulty functioning of the stimulation system or orientate better programming [14]. Regarding the autonomic function, a 24 h Holter can assist using spectral analysis. CHD is associated with a reduction in the sympathetic response (reduced LF—low frequency), as well as an overall decrease in autonomic function observed by the reduction of Standard deviation of sequential 5-min N-N interval means (SDANN). The increased rMSSD (square root of the mean of the square of the differences between adjacent normal RR intervals, in a time interval) and pNN50 (percentage of absolute differences in successive normal sinus values >50 ms) values reflect malfunction in their vagal tone.

### 2.3. Exercise Test

When available, exercise tests should be performed to understand the responses of Chagas disease patients to hemodynamic stress and to assess the functional capacity. The most important finding to observe on ECG during the exam are ventricular arrhythmias, which are independent predictors of mortality [1,15]. They indicate progression of CHD and worsening of cardiomyopathy. The chronotropic response and the heart rate in the first minute of recovery can show how sympathetic and parasympathetic act, since there is an autonomic dysfunction in Chagas disease patients. The assessment of functional capacity is important to guide professional and activity restrictions, to follow the worsening on function capacity and beginning of heart failure. In advanced heart failure patients, the exercise test can suggest a cardiac transplantation indication when the peak oxygen uptake ≤10 mL/kg/min. Finally, exercise test could also useful to access ST changes in differential diagnosis in patients with classical chest pain, to exclude coronary disease and to elucidate patient’s symptoms during exercise.

## 3. Echocardiography

Echocardiography has become the most common method to assess and follow up patients with CHD. The presence of echocardiographic abnormalities allows us to stage disease progression (Table 1). In early stages, echocardiography may demonstrate segmental wall motion abnormalities, particularly in the apex or basal segments of inferior and inferolateral walls, usually without associated obstruction in epicardial coronary arteries [16]. These lesions usually occur due to microcirculatory derangements. Diastolic dysfunction is also a common early finding.

The landmark lesions of CHD are LV apical aneurysms. In order to identify these aneurysms, it is important to use not only standard but also angulated views such as modified four- and two-chambers views, aiming posteriorly and avoiding foreshortening, dropout or near-field artifacts. Aneurysms are not limited to the apex or inferolateral walls, the most common sites, and may be associated to the presence of intraventricular mural thrombi. Right ventricular (RV) aneurysm is uncommon. The prevalence of LV aneurysm is inferior to 10% in asymptomatic patients but has been reported as superior to 50% in patients with more advanced stages, with moderate to severe LV global systolic dysfunction. Symptomatic patients may present regional wall motion abnormalities, LV or biventricular dilatation with diminished LV ejection fraction.

Echocardiograms should be performed at least once in every patient with positive serology and can be repeated each 3–5 years or anytime if ECG abnormalities are detected and should follow the European Association of Cardiovascular Imaging/American Society of Echocardiography task force’s recommendations on chamber quantification, LV function and morphology, RV function and valvular analysis [17].

Chamber volumes and global ventricular function should be evaluated by bidimensional (2D) echocardiography through biplane method of disks (Simpson’s) and tridimensional (3D) echocardiography, whenever possible. Tridimensional is more accurate than 2D echo for assessing LV volumes and ejection fraction (EF), particularly in the presence of wall motion abnormalities when there is a distorted LV geometry, as it allows visualization of cardiac chambers without geometric assumptions. It can also avoid foreshortening, being more accurate to detect the presence of aneurysms or thrombus. Regional wall motion abnormalities are most often located in the apex, inferior and inferolateral walls and should be assessed in multiple angles. Evaluation should include valvular structure and function, as functional mitral and tricuspid valve regurgitation are common in advanced cases with ventricular remodeling and valve annular dilation.

**Figure 1:**
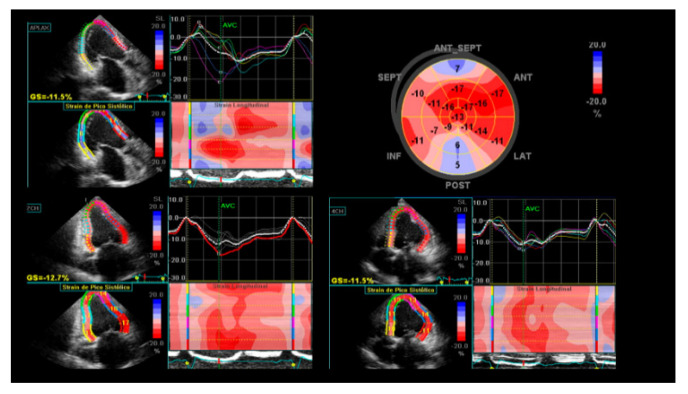
Global longitudinal strain of the left ventricle and strain curves depicting increased mechanical dispersion. Abnormal findings of left ventricular (LV) longitudinal strain in apical three-chamber view (**top left**); apical two-chamber view (**bottom left**); apical four-chamber view (**bottom right**); “Bull’s-eye” plot of strain values for each myocardial segment evidences anteroseptal and inferolateral akinesia represented by blue areas in the polar map (**top right**).

Strain is a measure of myocardial deformation, defined as the change of length of the myocardium that allows a more precise and quantitative measurement of the regional myocardial function, overcoming the subjective evaluation by conventional echocardiography. Strain obtained by speckle-tracking is the method of choice because it is not angle dependent. Regional strain is of particular interest in CHD due to the frequent segmental myocardial involvement (Figure 1), being particularly useful for increased recognition of subclinical myocardial dysfunction during indeterminate form of CHD [18,19]. Global longitudinal strain is the most validated method and is correlated with the amount of myocardial fibrosis, as shown is a study including 125 patients with Chagas disease, which found that longitudinal strain was reduced in the patients who had cardiac fibrosis on CMR, despite no significant difference in LV ejection fraction compared with patients without cardiac fibrosis [20].

Echocardiography may potentially be used to study ventricular dyssynchrony in CHD [21,22]. Duarte et al. [22] reported that the prevalence of interventricular dyssynchrony was 34% and of intraventricular dyssynchrony was 85%. However, dyssynchrony was not a strong predictor of clinical events, and the ECG remains the most important tool for indicating cardiac resynchronization therapy. Contrast echocardiography can be useful to enhance LV endocardial border and increase detection of aneurysms and thrombus [15].

Echocardiography is an inexpensive and readily available method, being very useful to stage and follow these patients, giving hemodynamic information that can be extremely helpful in advanced heart failure. On the other hand, the method is less sensitive to evaluate the presence of fibrosis and is not able to detect inflammation.

## 4. Computed Tomography

Cardiac computed tomography (CT) has an excellent negative predictive value to exclude coronary artery disease through coronary computed tomography angiogram (CTA), being extremely useful in patients with low to intermediate pre-test probability. Cardiac CT can also be used to plan electrophysiologic procedures, evaluate LV morphology, including the detection of regional wall motion abnormalities, apical aneurysms and intracardiac thrombi, and to calculate LV function in patients with difficult echocardiographic windows and contraindication to CMR, such as the presence of an implanted cardiac device [1,5].

## 5. Cardiovascular Magnetic Resonance

**Figure 2:**
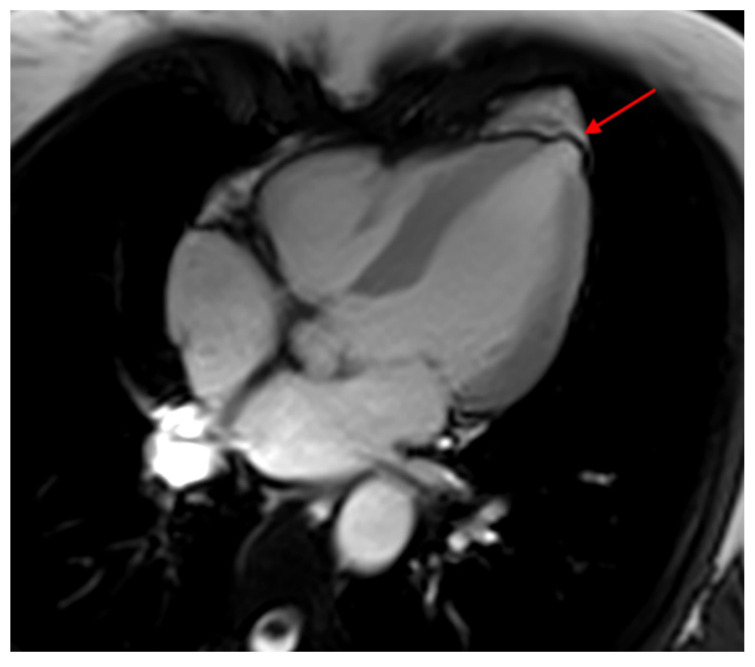
Cardiac magnetic resonance (CMR) four chamber image showing a typical left ventricular mamillar apical aneurysm (arrow). CMR: cardiac magnetic resonance.

CMR has a superior capability for anatomic and functional evaluation of cardiac chambers and measurement of right and left ventricular EF. LV systolic dysfunction is the strongest predictor of morbidity and mortality in CHD, and detection of subclinical dysfunction may orient therapeutic measures that would help in delaying the progression of the disease. Regional wall motion abnormalities, thrombus and aneurysms can be easily recognized (Figure 2).

Myocardial fibrosis is a histopathological finding common to several types of cardiovascular diseases and is associated with morbidity and mortality [23,24]. The mechanisms leading to fibrosis are variable and there may be an imbalance between collagen production and degradation or myocyte death [25].

The presence of fibrotic myocardial lesions is associated to reentrant circuits, which are the main pathophysiologic mechanism of ventricular tachyarrhythmias. Recent studies reveal that the degree of myocardial fibrosis increases progressively from the mildest to the most severe disease stages and late gadolinium contrast enhancement (LGE) evaluated on CMR is currently the best non-invasive method to assess it, being a marker of subclinical involvement with proven prognostic value [26,27,28]. The presence of scar by LGE is strongly associated with high risk of SCD [29].

**Figure 3:**
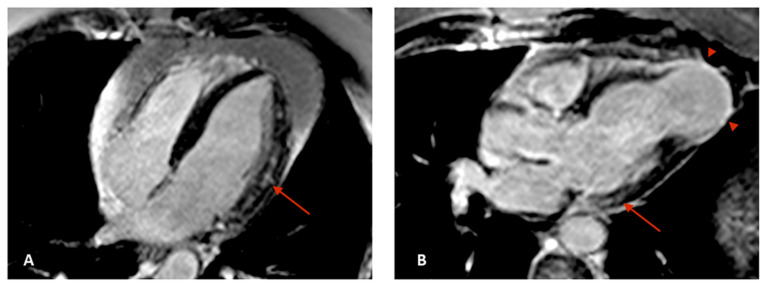
CMR images in four chamber (**A**) and three chamber (**B**) views showing meso-epicardial (arrows) and apical transmural (arrowheads) late gadolinium enhancement (LGE). CMR: cardiac magnetic resonance; LGE: late gadolinium enhancement.

Development of fibrosis in Chagas disease patients predominates in territories of distal circulation, particularly in apical and inferolateral regions, and the pattern of LGE is usually meso-epicardial or transmural (Figure 3), which differs from ischemic cardiomyopathies, in which LGE with a characteristic subendocardial fibrosis pattern matches a coronary territory distribution [11].

**Figure 4:**
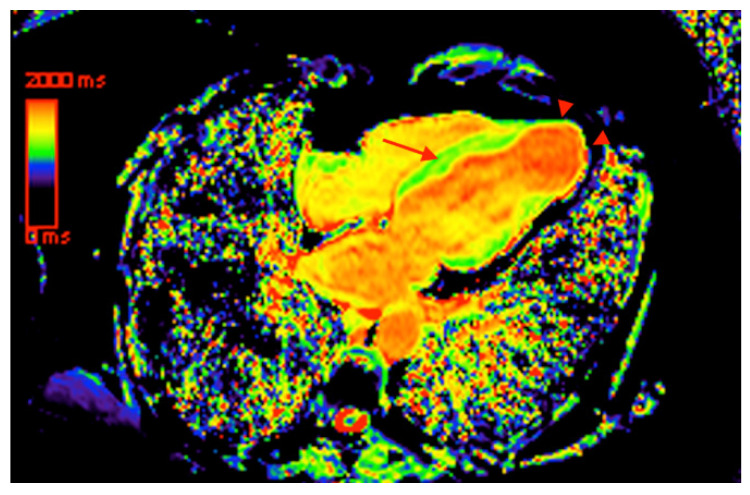
Four chamber image with color-coded native (non-contrast) T1 map, indicating the presence of mesocardial septal fibrosis (arrow) and apical transmural fibrosis (arrowheads). Increased native T1 is represented by yellow areas within the myocardium.

Nonetheless, LGE fails to account for interstitial and diffuse collagen distribution, which leads to underestimation of the total myocardial fibrosis mass [30]. The capability of tissue characterization with detection of myocardial edema and interstitial fibrosis has been recently reported in ischemic and non-ischemic cardiomyopathies and may be helpful in risk assessment [31]. New CMR techniques, such as native T1 mapping (Figure 4) and myocardial extracellular volume (ECV) calculation quantify diffuse myocardial fibrosis with close correlation with histological studies [32]. Avanesov et al. [31] reported that global ECV was superior to other CMR parameters, including LGE, to identify hypertrophic cardiomyopathy patients with syncope and non-sustained ventricular tachycardia, and presumably with increased risk of SCD. Future studies are necessary to evaluate the usefulness of these techniques in Chagas disease.

## 6. Nuclear Medicine

Patients with chronic CHD have an increased risk of SCD compared to other cardiomyopathies. The presence of fibrosis, dysautonomia and persistent cardiac inflammation could contribute to the risk of SCD, offering a substrate for ventricular tachycardia (VT). Molecular imaging with single photon emission computed tomography (SPECT) or positron emission tomography (PET) using a variety of radiotracers are valuable tools to identify changes that predispose to arrhythmia, such as hypoperfusion, inflammation and abnormal sympathetic innervation.

**Figure 5:**
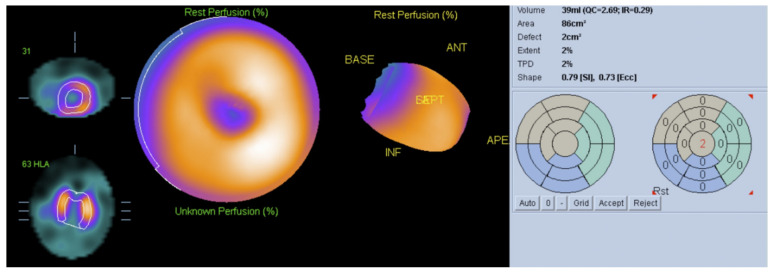
Automatic quantification (polar map) from rest 99m-Technetium sestamibi scintigraphy demonstrating reduced myocardial perfusion in apical region in a patient with a small aneurism of the left ventricular apex.

Myocardial perfusion imaging may be performed with SPECT or hybrid SPECT-CT equipment, using technetium-99m labeled radiopharmaceuticals such as tetrofosmin or sestamibi (Figure 5), or with PET and hybrid PET-CT using radiopharmaceuticals such as Rubidium-82. A disturbance due to microvascular ischemia participates in the mechanisms, causing myocardial injury and can occur in the early stages of CHD [33]. The use of hybrid SPECT-CT and PET-CT equipment allows attenuation correction, reducing artifacts.

**Figure 6:**
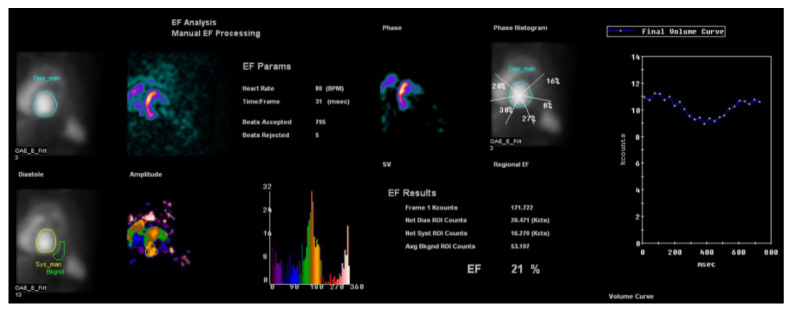
Multigated acquisition (MUGA) scan demonstrating reduced ejection fraction (EF = 21%) in a patient with Chagasic cardiomyopathy. This radionuclide ventriculography technique is a highly accurate test, used to determine the heart’s pumping function, and it shows substantial reproducibility and low intraobserver and interobserver variability.

It has been demonstrated that histogram bandwidth and phase-derived standard deviation may be analyzed in gated-SPECT myocardial perfusion imaging (Figure 6), which allows the assessment of intraventricular synchronism. In comparison to echocardiography, nuclear medicine techniques have the advantage of lower interobserver variability and higher reproducibility. Peix et al. [34] studied myocardial perfusion and intraventricular synchronism in the indeterminate phase of Chagas disease which presented tissue Doppler imaging-derived strain. A total of 8% of subjects had perfusion defects, 28% had a post-stress LVEF reduction of >5%. The authors found that histogram bandwidth and phase-derived standard deviation indicated a significant difference between post-stress and rest. In these cases, there was a minor dyssynchrony at rest that normalized at post-stress.

**Figure 7:**
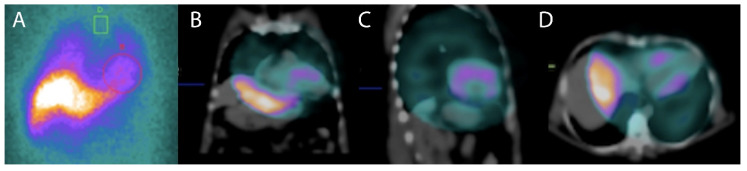
A 123I-MIBG scintigraphy planar scintigraphy in anterior view at 3 h (**A**) to evaluate the sympathetic innervation a patient with Chagasic cardiomyopathy and ventricular arrhythmia. Heart to mediastinum ratio at 3 h was reduced and radiotracer washout was increased. Single photon emission computed tomography (SPECT)-computed tomography (CT) images in coronal (**B**), sagittal (**C**) and transversal (**D**) planes demonstrated reduced cardiac uptake of 123I-MIBG in apical, inferior and lateral regions indicating sympathetic denervation in these areas and a worse prognosis.

Abnormalities in cardiac sympathetic innervation may play a central role in the mechanism triggering serious ventricular arrhythmias and SCD in patients with non-ischemic cardiomyopathy. The work of Miranda [35] showed that regional myocardial sympathetic denervation assessed with Iodine-123 metaiodobenzylguanidine (123I-MIBG) scintigraphy is associated with sustained ventricular tachycardia in CHD, concluding that viable although denervated myocardial areas were associated to the genesis of sustained ventricular arrhythmias (Figure 7). Cardiac autonomic sympathetic modulation detected with 123I-MIBG SPECT can also be affected in subjects with early forms of CHD with preserved ventricular function, as reported by Landesmann [36]. Recently, Gadioli [37] used 123I-MIBG SPECT to assess the extension of the sympathetic denervation, and showed its association with the severity of the ventricular arrhythmias. It is necessary to design prospective studies for the assessment of cardiac sympathetic innervation. Results could be used for risk stratifications of sudden death, which would be extremely useful if we consider the lack of consensus related to the stratification and primary prevention of sudden cardiac death in patients with Chagas disease.

The presence of persistent cardiac inflammation may also be related to ventricular tachycardia and the risk of sudden death. Persistent subclinical inflammation in areas adjacent to fibrotic regions may act as a trigger for reentry in the presence of fibrosis. In addition, active inflammation may increase the automaticity within inflamed areas. Radionuclide imaging can allow the identification of areas of inflammation of the myocardium in patients with non-ischemic cardiomyopathies [38].

^18^F-fluorodeoxyglucose (^18^F-FDG) is a glucose analogue being physiologically captured by cardiomyocytes which can be used to evaluate the pathologic uptake by inflammatory cells. Recently, the use of ^18^F-FDG PET-CT has been studied in patients with sarcoidosis, demonstrating that the presence of focal perfusion defects and increased FDG uptake in patients with suspected cardiac sarcoidosis identified subjects at higher risk of death or ventricular tachycardia [39].

The use of PET-CT in Chagas disease has not yet been systematically studied. There are several similarities between Chagas disease and sarcoidosis, so the use of this imaging technique could potentially be useful in assessing the presence of inflammation and prognosis. Both are associated with changes in the conduction system and complex ventricular arrhythmias and with the presence of meso-epicardial fibrosis.

**Figure 8:**
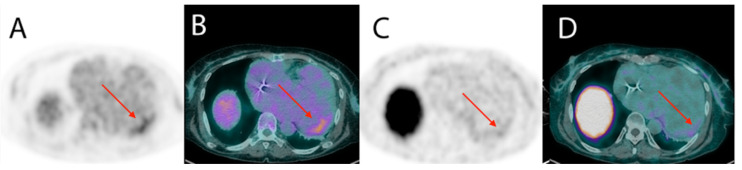
Increased cardiac volume in a patient with Chagasic cardiomyopathy. Mild increase in uptake of 18F-FDG (**A**,**B**) and of 68Ga-DOTATOC (**C**,**D**) in positron emission tomography (PET)-CT images was restricted to the basal anterolateral segment, indicating the presence of inflammation in this segment.

Currently, patients with chronic Chagas disease are not routinely evaluated for the presence of inflammation. Three case reports have described increased uptake of 18F-FDG PET in patients with Chagas disease and ventricular tachycardia, suggesting that the presence of inflammation could be involved in the genesis of the arrhythmia [40,41]. Recently, the use of new radiotracers that are not physiologically accumulated in the normal myocardium, including somatostatin receptor imaging radiopharmaceuticals such as gallium-68 (^68^Ga) labeled-DOTATOC may overcome some limitations of the ^18^F-FDG, as has been reported for cardiac sarcoidosis and CHD [42,43], as illustrated in Figure 8.

PET-CT has the advantage of a higher spatial resolution when compared to scintigraphy and, unlike CMR, it can be used in patients with implantable cardiac devices. Better understanding of the role of inflammation in these patients may provide novel treatment strategies, such as localization of the anatomic substrate before ablation of VT, and improved risk stratification and orientation about primary prevention with ICD implantation.

**Figure 9:**
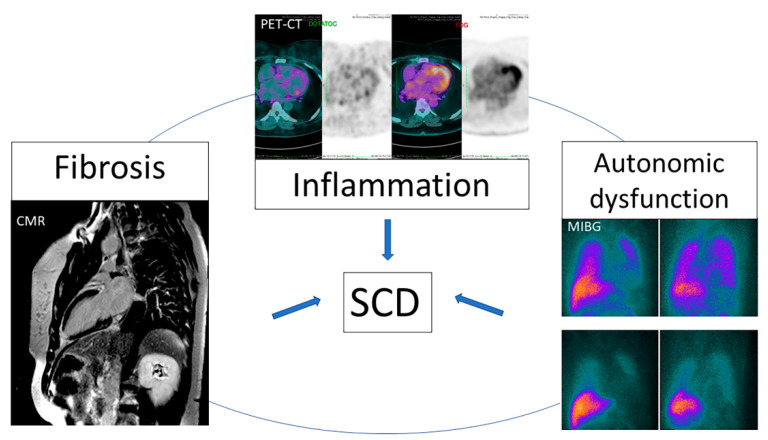
New imaging modalities can detect abnormalities involved in the genesis of ventricular arrythmia. SCD: Sudden cardiac death.

Unfortunately, despite its significant mortality, there is no clear recommendation for early ICD implantation in patients with CHD in the current guidelines. Ideally, the risk of SCD should be evaluated in earlier stages of the disease. New imaging parameters that are useful to identify factors involved in the genesis of arrhythmia such as fibrosis, inflammation and dysautonomia (Figure 9) can be a promising strategy.

## 7. Conclusions

Fibrosis, inflammation and dysautonomia are involved in the genesis of ventricular arrhythmia in CHD. Due to the risk of sudden death, it is important that such events are early identified, so that SCD preventive measures can be taken. Myocardial fibrotic areas leads to a macro reentry circuit that triggers ventricular tachycardia and CMR is an accurate method to identify these areas. The presence of inflammatory process, detected through PET-CT with FDG or DOTATOC, may be involved in the genesis of the arrhythmia in CHD. Finally, studies of myocardial dysautonomia identified using myocardial SPECT-CT with MIBG have reported an association between denervated areas and ventricular electrical instability, even in the presence of viable myocardium. Echocardiography, despite not being sensitive enough to identify fibrosis and denervation, can indicate severe ventricular dysfunction, which is also associated with an increased risk of sudden death. Other non-invasive tests such as ECG, 24 h Holter and exercise test can identify both dysautonomia, and the presence of ventricular electrical instability, demonstrating an increased risk of sudden death in CHD patients. Thereby, traditional non-invasive diagnostic methods associated to new image parameters obtained through CMR, SPECT-CT and PET-CT can improve risk stratification of SCD and play a promising role in the implementation of primary preventive strategies.

## Figures and Tables

**Table 1:** Definition and progression of chronic Chagas heart disease.

Stage	ECG	Echocardiogram	Heart Failure
A	abnormal	Normal	Absent
B1	abnormal	abnormal, LVEF ≥45%	Absent
B2	abnormal	abnormal, LVEF <45%	Absent
C	abnormal	Abnormal	Reversible
D	abnormal	Abnormal	Refractory

Adapted from: Xavier SS et al. [7]. LVEF = left ventricle ejection fraction.
